# Early In-Hospital Mortality following Trainee Doctors' First Day at Work

**DOI:** 10.1371/journal.pone.0007103

**Published:** 2009-09-23

**Authors:** Min Hua Jen, Alex Bottle, Azeem Majeed, Derek Bell, Paul Aylin

**Affiliations:** 1 Dr Foster Unit at Imperial College, Department of Primary Care and Social Medicine, Imperial College London, London, United Kingdom; 2 Department of Primary Care and Social Medicine, Imperial College London, London, United Kingdom; 3 Department of Medicine, Imperial College London, Chelsea and Westminster Campus, London, United Kingdom; Copenhagen University Hospital, Denmark

## Abstract

**Background:**

There is a commonly held assumption that early August is an unsafe period to be admitted to hospital in England, as newly qualified doctors start work in NHS hospitals on the first Wednesday of August. We investigate whether in-hospital mortality is higher in the week following the first Wednesday in August than in the previous week.

**Methodology:**

A retrospective study in England using administrative hospital admissions data. Two retrospective cohorts of all emergency patients admitted on the last Wednesday in July and the first Wednesday in August for 2000 to 2008, each followed up for one week.

**Principal Findings:**

The odds of death for patients admitted on the first Wednesday in August was 6% higher (OR 1.06, 95% CI 1.00 to 1.15, p = 0.05) after controlling for year, gender, age, socio-economic deprivation and co-morbidity. When subdivided into medical, surgical and neoplasm admissions, medical admissions admitted on the first Wednesday in August had an 8% (OR 1.08, 95% CI 1.01 to 1.16, p = 0.03) higher odds of death. In 2007 and 2008, when the system for junior doctors' job applications changed, patients admitted on Wednesday August 1^st^ had 8% higher adjusted odds of death than those admitted the previous Wednesday, but this was not statistically significant (OR 1.08, 95% CI 0.95 to 1.23, p = 0.24).

**Conclusions:**

We found evidence that patients admitted on the first Wednesday in August have a higher early death rate in English hospitals compared with patients admitted on the previous Wednesday. This was higher for patients admitted with a medical primary diagnosis.

## Introduction

Despite the commonly held assumption that early August is an unsafe period to be admitted to hospital in England as residency starts the first Wednesday of August [1], previous work shows little evidence of an excess in deaths in the first week of August, when newly qualified junior doctors start work [2]. A limitation of a previous study on this topic [2] was that the authors did not have any data on admissions and were only able to examine numbers of deaths rather than mortality rates. The introduction of the European working time directive [3], [4] that has had a direct impact on medical staffing in all European Union health systems and controversy over a computerised recruitment system (Medical Training Application Service or MTAS) in 2007 has resurrected these concerns [5].

This study is the largest of its kind and has international implications. A similar effect has been recorded in the US (known as the “July phenomenon” [6]). Most studies on the July phenomenon have not shown a statistically significantly higher mortality risk, but these have only included one hospital or few hospitals from a region [6], [7], [8], [9], [10]. They are therefore limited in terms of statistical power and unable to detect small differences in mortality. By examining more hospitals over a longer period of time, Huckman and Barro in 2005 [11] found a small relative increase in mortality annually for US teaching hospitals over non-teaching hospitals from 1993 to 2001.

We investigated whether there are higher death rates in the week following the first Wednesday in August in England using routinely collected hospital administrative data for the past nine years. We also examined the first Wednesday in August in 2007 and 2008 separately because of concerns about the impact of MTAS and its replacement on the provision of inpatient care in National Health Service (NHS) hospitals in England. During these last two years, all training posts (including specialist registrars) changed over on the same date. The magnitude of the effect of such a transition has implications for hospital managers, senior physicians and policy makers in their annual planning.

## Methods

The National Health Service (NHS) in England is a publicly funded health service with universal access based on patients' needs. There are 1,600 NHS hospitals and specialist care centres under 175 acute and 60 mental health NHS trusts (http://www.nhs.uk/aboutnhs/HowtheNHSworks/Pages/NHSstructure.aspx). Hospital Episode Statistics (HES) [12], the hospital administrative dataset in England, comprises data gathered locally through each hospital's Patient Administration System or Hospital Information System. It covers all inpatient and day-case activity delivered by NHS hospitals in England with over 14 million records collected annually. The dataset contains over 300 fields and includes age, gender, admission method, fourteen diagnosis fields, twelve operation fields, length of stay, waiting times, ethnic group and method of discharge (including in-hospital death). We have approval from St. Mary's Hospital Local Research Ethics Committee to use routinely collected hospital administrative data for research. Also, we have been granted permission by the Patient Information Advisory Group under Section 251 of the NHS Act 2006 (originally enacted under Section 60 of the Health and Social Care Act 2001) to use routinely collected hospital administrative data to provide measures of quality of delivery of healthcare by provider.

We examined HES for the period from 2000 to 2008 (the Secondary Uses Service [13] was used as the source for these data for 2006, 2007 and 2008). The basic unit of the dataset is the consultant episode, covering the continuous period of time during which a patient was under the care of one consultant. Episodes of care were linked into admissions (spells) and those ending in transfer to another hospital were linked together to avoid multiple counting. We derived the main diagnosis from the primary diagnosis of the first episode of care. If that diagnosis was vague or non-specific, then the primary diagnosis in the subsequent episode of care (if present) was used. All admissions were coded to one of 259 Clinical Classification System (CCS) diagnostic groups [14]. We carried out the analysis in two parts.

Firstly, examining data for 2000 to 2008, we established two cohorts: one cohort consisting of patients admitted as an emergency (the patient's admission to hospital was unplanned/non-elective) on the last Wednesday of July and the other comprising patients admitted as an emergency on the first Wednesday of August, with each cohort followed up for one week. We only included acute hospitals (at 175 acute trusts) that take on trainee doctors on the first Wednesday of August each year. If, by the end of the following Tuesday, a patient had died in hospital, we counted them as a death; otherwise we presumed them to have survived. We calculated the odds of death in admissions occurring on the week after the first Wednesday in August compared with those on the week before, adjusted for age (20 groups: <1 year, 1–4, 5–9, and five-year bands up to 90+), sex, area-level socio-economic deprivation (quintile of Carstairs index [15] of deprivation), year (NHS financial year of discharge, from 1^st^ April each year to the 31^st^ March the next year), week and co-morbidity (using the Charlson index [16] of co-morbidity, ranging from 0 to 6+). We created our model by entering all covariates at once. A previous study showed that models using HES data can have good discrimination (as assessed by the receiver operating curve c statistic) and calibration (using standardised residuals measuring goodness-of-fit) [17]. P values less than 0.05 were considered statistically significant. Secondly, we looked at the 2007 and 2008 changeover separately from other years because of the controversy over MTAS [18], [19].

We also assessed whether patients are different between the two weeks in their primary diagnosis group for all years combined and year 2007 and 2008 only. As there can be very few patients in some diagnosis groups, we have divided the 259 groups into three clinical categories: medical, surgical and neoplasms as in a previous analysis [20]. The two weeks were first compared using chi-squared tests. We then calculated the odds of death (adjusted for casemix) separately for each clinical category.

Patients admitted to the same hospital will all be subject to similar hospital-level influences and will therefore have more similarity in their outcomes than similar patients admitted to another hospital. Also, some patients are admitted more than once. To account for this ‘clustered’ data structure and as our outcome is a binary variable, we used multilevel logistic regression [21] and carried out the estimation using the MLwiN program (version 2.10) and MCMC estimation procedures [22].

## Results

In the two cohorts derived from the two dates of admission, between 2000 and 2008, there were a total of 299,741 admissions (151,844 admitted on the last Wednesday in July and 147,897 admitted on the first Wednesday in August) and 4,409 deaths in hospital within a week of admission (2,182 for the former week and 2,227 for the latter week). In the years 2007 and 2008, there were a total of 73,857 admissions (37,729 admitted on Wednesday 25^th^ July and 36,128 admitted on Wednesday 1^st^ August) and 986 deaths (491 for the former week and 495 for the latter week) in hospital within a week of admission. Throughout the period, there were fewer emergency admissions on the first Wednesday in August every year than on the last Wednesday in July. There was no clear pattern to these differences, however, with the greatest difference present in 2007. There was little difference between the two weeks in terms of deprivation and comorbidity, with a slightly higher proportion of admissions in the most deprived quintile in the first week of August for all nine years combined. In 2007 and 2008, there was little difference between patients admitted on the last Wednesday of July and those admitted on the first Wednesday in August (Table 1).

**Table 1:** Descriptive statistics for casemix measures for the two cohorts: emergencies admitted on the last Wednesday in July versus the first Wednesday in August for the years 2000 to 2008 combined and 2007 and 2008 only.

	Emergency admissions on the last Wed in July	Emergency admissions on the first Wed in August	Comparison between the two weeks
	2000 to 2008	combined	2007 & 2008	only	2000 to 2008	combined	2007 & 2008	only	2000 to 2008 combined	2007 & 2008 only
Variables	Admissions (% of total)	Deaths	Admissions (% of total)	Deaths	Admissions (% of total)	Deaths	Admissions (% of total)	Deaths	P value	P value
**Total**	151844	2182	37729	491	147897	2227	36128	495		
**Year:** 2000	14899 (9.81)	243	.	.	14676 (9.92)	254	.	.	0.09	0.66
2001	15122 (9.96)	211	.	.	14712 (9.95)	221	.	.		
2002	15482(10.20)	247	.	.	15089 (10.20)	234	.	.		
2003	15844 (10.43)	241	.	.	15706 (10.62)	288	.	.		
2004	16893 (11.13)	248	.	.	16545 (11.19)	273	.	.		
2005	17463 (11.50)	243	.	.	17411 (11.77)	234	.	.		
2006	18412 (12.13)	257	.	.	17630 (11.92)	225	.	.		
2007	18726 (12.33)	246	18726 (49.63)	246	17745 (12.00)	255	17745 (49.12)	255		
2008	19003 (12.51)	245	19003 (50.37)	245	18383 (12.43)	240	18383 (50.88)	240		
**Age:** = <15	36027 (23.71)	114	8751 (23.19)	35	34884 (23.56)	108	8349 (23.11)	29	0.48	0.91
20–60	74593 (49.09)	315	18644 (49.42)	62	72984 (49.30)	366	17912 (49.58)	77		
> = 65	41332 (27.20)	1753	10334 (27.39)	394	40166 (27.13)	1753	9867 (27.31)	389		
**Gender:** Male	58843 (38.72)	1062	14556 (38.58)	237	57014 (38.57)	1073	14099 (39.03)	254	0.40	0.22
Female	93109 (61.28)	1120	23173 (61.42)	254	90930 (61.43)	1154	22029 (60.97)	241		
**Charlson score**: 0	115774 (76.25)	563	28003 (74.22)	119	113380 (76.66)	607	26970 (75.65)	132	0.03	0.33
1	20239 (13.33)	637	5596 (14.30)	133	19429 (13.14)	680	5177 (14.33)	136		
2	8874 (5.84)	415	2336 (6.19)	86	8494 (5.74)	395	2149 (5.95)	91		
3	3316 (2.18)	229	941 (2.49)	68	3233 (2.19)	251	899 (2.49)	72		
4	1191 (0.78)	96	360 (0.95)	30	1047 (0.71)	83	316 (0.87)	19		
5	1795 (1.18)	176	504 (1.34)	40	1659 (1.12)	132	429 (1.19)	23		
> = 6	655 (0.43)	65	189 (0.50)	15	655 (0.44)	76	188 (0.52)	22		
**Deprivation quintile**: 1(least deprived)	21188 (13.94)	280	5215 (13.82)	58	20606 (13.92)	310	5032 (13.93)	64	0.06	0.45
2	24175 (15.91)	405	5939 (15.74)	91	23426 (15.82)	415	5687 (15.74)	97		
3	27248 (17.93)	432	6691 (17.73)	102	26205 (17.70)	476	6417 (17.96)	116		
4	31020 (20.41)	507	7703 (20.42)	120	30061 (20.31)	472	7262 (20.10)	96		
5 (most deprived)	37598 (24.74)	482	9423 (24.98)	99	36921 (24.94)	474	8955 (24.79)	101		
6 (unknown)	10723 (7.06)	76	2758 (7.31)	21	10815 (7.31)	80	2775 (7.68)	21		
**Diagnosis groups:** Medical	129136 (85.05)	1652	32184 (85.30)	386	125378 (84.77)	1713	30687 (84.94)	384	0.05	0.56
Surgical	18330 (12.07)	216	4593 (12.17)	52	18381 (12.43)	217	4575 (12.66)	57	0.99	0.62
Neoplasms	4378 (2.88)	313	952 (2.53)	53	4138 (2.80)	294	866 (2.40)	54	0.92	0.55

For all years combined, we found a small non-significant difference in the crude odds ratio of death for the week from the first Wednesday in August compared with the week from the last Wednesday in July (OR 1.05, 95% CI 0.99 to 1.11, p = 0.12). Adjustment for year, gender, age group, socio-economic deprivation and co-morbidity led to a 6% higher odds ratio of death for the second week (OR 1.06, 95% CI 1.00 to 1.13, p = 0.05). This effect did not differ significantly from year to year (there was no significant interaction between week and year, p = 0.32). Analysing patients by their clinical diagnosis category, we did not find statistically significant differences (p>0.10) between the two weeks for surgical patients (36,711 admissions; 12.3% of all admissions) and for patients with neoplasms (8,516 admissions; 2.8% of all admissions). For the medical category, there was a weakly statistically significant difference (p = 0.05) between the two weeks (254,514 admissions; 84.9% of all admissions) with a crude odds ratio of death for the week from the first Wednesday in August compared with the week from the last Wednesday in July of 1.07 (95% CI 1.00 to 1.15). Adjustment for year, gender, age group, socio-economic deprivation and co-morbidity led to an 8% higher odds ratio of death for the second week (OR 1.08, 95% CI 1.01 to 1.16, p = 0.03) (Table 2).

**Table 2:** Odds of death in admissions occurring on the week after the first Wednesday in August compared with the week from last Wednesday in July (as the base) for the years 2000 to 2008 combined and 2007 and 2008 only using multilevel logistic regression.

	2000 to 2008 combined		2007 and 2008 only	
Models	Odds ratio (P value)	95% CI	Odds ratio (P value)	95% CI
**All diagnosis**				
No covariates included	1.05 (0.12)	0.99–1.11	1.05 (0.42)	0.93–1.19
All covariates included	1.06 (0.05)	1.00–1.13	1.08 (0.24)	0.95–1.23
**Reallocate by primary diagnosis**				
**Medical**				
No covariates included	1.07 (0.05)	1.00–1.15	1.04 (0.56)	0.91–1.20
All covariates included	1.08 (0.03)	1.01–1.16	1.06 (0.40)	0.93–1.21
**Surgical**				
No covariates included	1.00 (0.99)	0.83–1.21	1.10 (0.62)	0.76–1.61
All covariates included	1.01 (0.96)	0.83–1.22	1.11 (0.60)	0.75–1.64
**Neoplasm**				
No covariates included	0.99 (0.92)	0.84–1.17	1.13 (0.55)	0.76–1.66
All covariates included	0.99 (0.89)	0.85–1.16	1.12 (0.58)	0.75–1.66

For the years 2007 and 2008 only, the odds of death for patients admitted on the first Wednesday in August was 5% higher than for patients admitted on the last Wednesday in July but this difference was not statistically significant (OR 1.05, 95% CI 0.93 to 1.19, p = 0.42). After adjusting for covariates, the odds of dying was still not significantly higher (OR 1.08, 95% CI 0.95 to 1.23, p = 0.24). We did not find significant differences in the crude or adjusted odds ratio of death between the week beginning the first Wednesday in August and the week beginning the last Wednesday in July for patients admitted with any primary diagnosis (p>0.10) (Table 2).

The most common diagnoses associated with death in our data for the first week of August were: pneumonia (excluding cases caused by tuberculosis or sexually transmitted disease); cancer of bronchus and lung; and aortic, peripheral, and visceral artery aneurysms.

## Discussion

In this large study of 299,986 emergency admissions during 2000–2008, we found overall a small but significant 6% higher odds of death for all patients and a statistically significant 8% higher odds of death for medical patients in the week following the first Wednesday in August than in the week following the last Wednesday in July. The first Wednesday in August is the period when all UK hospitals plan and undertake the changeover of junior doctors. Shuhaiber et al [23] assessed the effect of turnover of cardiothoracic resident surgical staff on cardiac surgical outcomes during financial years 1996 to 2006 at a single institution and found a 30% higher risk of in-hospital mortality after complex cardiac operations, but not after CABG alone, for the month after turnover than in the month before. We did not see this effect for surgical patients as a group nor for patients admitted with malignancy. It may be that some operations might be more influenced by the turnover than others, but this was not our primary hypothesis.

We restricted our analysis to include only emergency admissions to avoid the potential bias of a difference in planned admissions between the two cohorts due to administrative pressures. Hospitals are likely to reduce their elective cases in anticipation of the holiday period and to accommodate the potential disruption of the junior doctor changeover. Patients admitted as an emergency are the largest group of patients admitted to most hospitals and are unwell requiring care at the time of admission. The short follow-up period was chosen to avoid any seasonal effects that might complicate our analysis if a longer period of time had been taken and late deaths may not reflect initial management and care. Nafsi et al [24] suggest a week's follow-up to best capture errors caused by failure of training or inadequate supervision, although Lu et al [25] monitored deaths within 24 hours as early mortality in the emergency department. In that setting, most of the early deaths are attributable to the natural course of the patient's disease. However, Lu et al [25] found that 25.8% of all deaths following emergency admission were preventable.

In both our analyses, there appeared to be no meaningful difference in casemix between the two cohorts, as shown by the small changes in the odds ratio following adjustment for these factors. However, there may have been other factors (such as severity of disease) that we were not able to adjust for and that may have accounted for the difference in mortality between the two groups. Multilevel modelling can separate the variation in mortality due to casemix variables from the unobserved variability due to hospitals. The latter was relatively wide and remained substantial (49% of the total variation) and significant when taking account of casemix variables. This strongly suggests that casemix variables cannot explain away all the differences and that there are other unobserved factors that need to be explored. It would have been of interest to further stratify the three clinical categories by specialty or even diagnosis to examine casemix further. However, small numbers mean that it becomes increasingly difficult to stratify beyond the three clinical subgroups used in this study. As we found previously [20] when comparing overall mortality by day of the week, deaths are largely in the medical subgroup.

We had fewer emergency admissions every year for the week following the first Wednesday of August than the week before across the whole study period. The admission threshold for an unplanned (emergency) admission varies from trust to trust, and might depend on factors such as bed availability and doctor confidence, as well as on the illness of the patients. There were around 4,000 fewer patients in total admitted on the August Wednesday than the July Wednesday, but the reasons are unclear.

National policy designed to support trainee induction and support has set the changeover date for the majority of junior doctors as the first Wednesday in August for starting new posts. Other factors could influence clinical outcomes in addition to new medical staffing, including the potential impact of the induction process itself. Equally, some but not all hospitals will ensure more senior cover is available over these periods. In this study we could not address these factors.

### Conclusion

We have found that patients admitted on the first Wednesday in August have a higher death rate than those admitted on the last Wednesday in July in hospitals in England. There was also a statistically significantly higher death rate for medical patients that was not evident for surgical admissions or patients with malignancy. If this is due to the changeover of junior hospital staff, then this has potential implications not only for patient care, but for NHS management approaches to delivering safe care. We suggest further work to look at other measures such as patient safety, quality of care, process measures or medical chart review [26] to identify preventable deaths rather than overall early mortality to further evaluate the effect of junior doctor changeover.
